# A novel echocardiographic approach indicates disease severity in pediatric pulmonary hypertension

**DOI:** 10.1111/ped.14163

**Published:** 2020-04-17

**Authors:** Martin Koestenberger, Alexander Avian, Massimiliano Cantinotti, Katharina Meinel, Georg Hansmann

**Affiliations:** ^1^ Division of Pediatric Cardiology Department of Pediatrics Medical University Graz Graz Austria; ^2^ Institute for Medical Informatics, and Statistics and Documentation Medical University Graz Graz Austria; ^3^ Fondazione CNR‐Regione Toscana G. Monasterio Massa and Pisa Italy; ^4^ Department of Pediatric Cardiology and Critical Care Hannover Medical School Hannover Germany

**Keywords:** New York Heart Association Functional class, pulmonary hypertension, tricuspid regurgitation velocity/ tricuspid annular plane systolic excursion ratio

**Highlights**

- We hypothesized that the TRV/TAPSE ratio reflect both the patients’ functional capacity and the hemodynamic performance of the individual RV‐ pulmonary artery (PA) unit.
- The novel TRV/TAPSE ratio correlated positively and strongly with NYHA FC, thus highlighting its clinical usefulness in future PH diagnosis.

Echocardiography is an easily available and, thus, preferred imaging modality to detect systolic right ventricular (RV) dysfunction, although quantification of any such cardiac impairment remains challenging. It has been previously reported that tricuspid annular plane systolic excursion (TAPSE) can be used for risk stratification of children with pulmonary hypertension (PH).^1^ In recent years, TAPSE has evolved into one of the major clinical indicators of systolic longitudinal RV function in pediatric PH.^2^ However, overreliance on a single variable has been proven to be an inadequate strategy for diagnosis or monitoring, so that current RV function research in PH patients follows multi‐parametric approaches that include TAPSE.^2^ We hypothesized that tricuspid regurgitation velocity (TRV), as an indicator of pulmonary artery pressure when combined with the TAPSE as the TRV/TAPSE ratio, reflects both the patients’ functional capacity and the hemodynamic performance of the individual RV to the pulmonary artery (PA) unit. Consequently, we investigated this ratio in a mid‐sized cohort of children and adolescents with PH and its possible association with hemodynamic and clinical variables, such as the pulmonary vascular resistance index (PVRi), the systolic pulmonary artery pressure/systolic systemic arterial pressure (sPAP/sSAP) ratio, and New York Heart Association Functional class (NYHA FC).

We investigated 47 children and adolescents with PH (median age: 7.2 years, range, 4.2 months−18.0 years; 46.8% female). NYHA FC was determined by two independent pediatric cardiologists. At enrollment, all patients were clinically stable without any change of medication in the preceding 12 weeks. A TRV >2.8 m/s was considered as the cut‐off value to define elevated PAP in the absence of RV outflow tract obstruction. All children with PH underwent cardiac catheterization. PH was defined according to the most recent 2018 World Symposium of Pulmonary Hypertension: a mean pulmonary artery pressure (mPAP) of >20 mm Hg at rest, a pulmonary arterial wedge pressure of ≤15 mm Hg, and a PVRi of >3 WU x m^2^.^3^ In those patients with PH (TRV >2.8 m/s), PAP and PVRi were invasively determined. TAPSE was measured as previously reported,^1^ and subsequently, the TRV/TAPSE ratio (m/s:cm) was calculated.

Group comparisons for continuous variables were analyzed using the Kruskal‐Wallis H‐test or ANOVA, as appropriate. Afterwards, a post hoc test (Mann‐Whitney *U *test or *t*‐test) for the comparison of the groups to each other were performed. Data are presented as median and interquartile range (IQR), unless otherwise stated. Age‐specified TAPSE *z*‐scores were calculated according to the reference values provided by Koestenberger *et al*.^4^ A *P*‐value < 0.05 was considered significant. 

This study complied with the institutional guidelines related to patient confidentiality and research ethics, and received institutional review board approval (Medical University Graz, Austria. EK Nr.: 31‐339 ex 18/19). Median TRV was 4 m/s (IQR: 3.5–4.3 m/s) and median PVRi was 6.2 WUxm2 (IQR: 3.7–9.2). The age‐specific TAPSE *z*‐score had a median of −2.79 (IQR: −7.37 to −2.39). The TRV/TAPSE ratio (m/s:cm) ranged from 1.53–8.40 (median: 2.64, IQR: 2.24–3.46) in our patients (Table S1). With worsening NYHA FC, the PVRi increased (NYHA FC 1: PVRi 3.4 [2.8–4.1]; NYHA FC 2: PVRi 6.6 [4.9–7.6]; NYHA FC 3: PVRi 13.1 [9.2–17.8] WUxm2;* P* < 0.001); in contrast TAPSE *z*‐scores (NYHA FC 1: −2.1 [−3.0 to −0.7]; FC 2: −2.8 [−3.7 to −2.1]; FC 3: −4.1 [−5.9 to −3.3]; *P *= 0.001) decreased. With worsening NYHA FC, the TRV/TAPSE ratio (FC 1: 2.11 m/s:cm [1.79–2.58]; FC 2: 2.64 m/s:cm [2.51–3.08]; FC 3: 3.87 m/s:cm [3.52–4.45]; *P *< 0.001) increased (Fig. 1a). We also divided the PH patients in groups: Group 1 (mild PH), sPAP/sSAP ratio of <0.5; Group 2 (moderate PH), 0.5–0.8; and Group 3 (severe PH), sPAP/sSAP ratio >0.8. By sPAP/sSAP ratio, the median (IQR) TRV/TAPSE ratio was: PH severity Group 1, TRV/TAPSE ratio 1.76 m/s:cm (1.71–2.10); PH severity Group 2, TRV/TAPSE ratio 2.60 m/s:cm (2.2–2.89); and PH severity Group 3, TRV/TAPSE ratio 3.47 m/s:cm (2.90–4.17); *P *< 0.001 (Fig. 1b).

**Figure 1:**
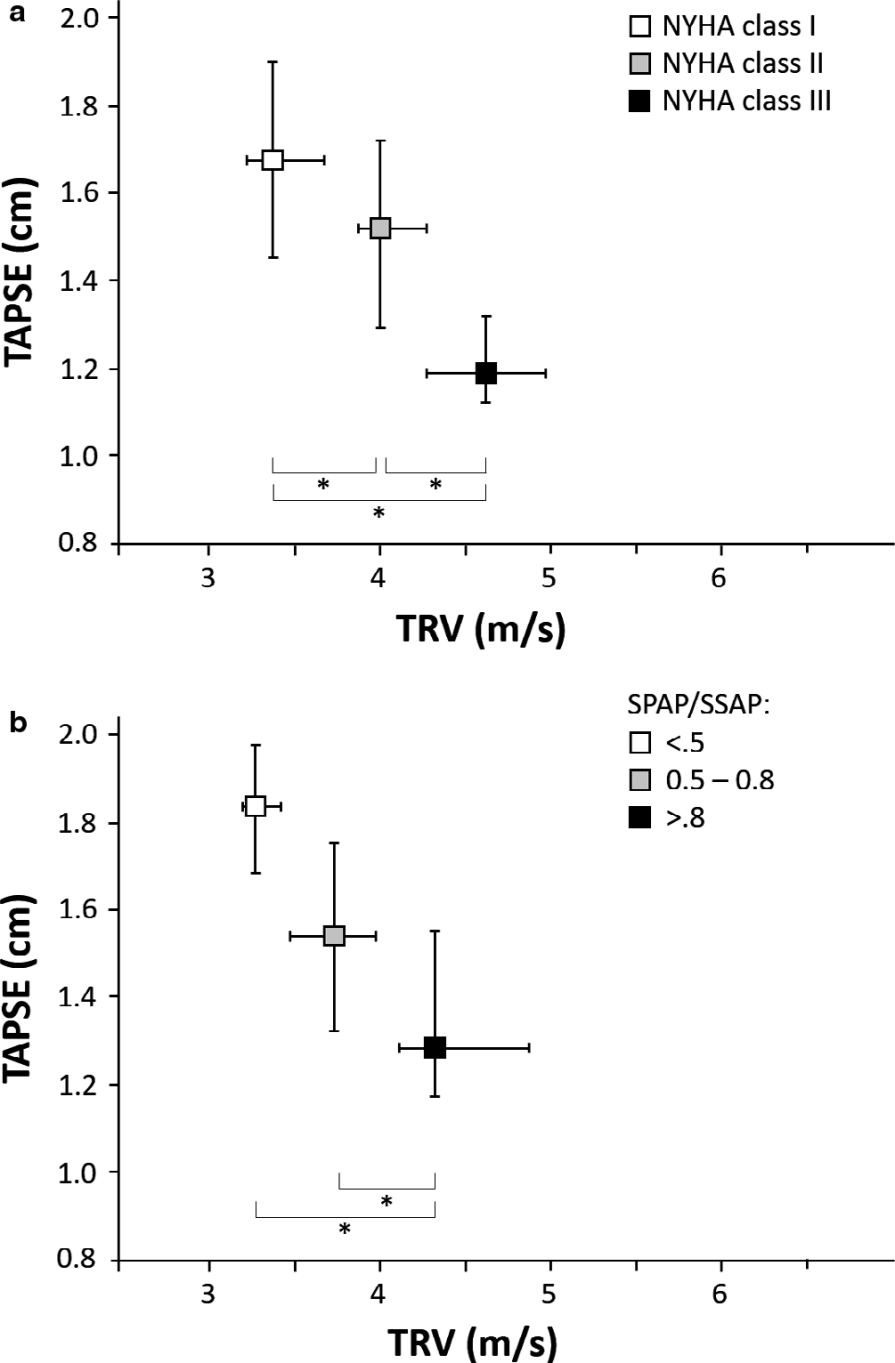
(a) Median tricuspid regurgitation velocity (TRV) to tricuspid annular plane systolic excursion (TAPSE) ratio distribution according to New York Heart Association Functional class (NYHA FC)/modified Ross score. (b) Median TRV to TAPSE ratio distribution according to sPAP, systolic pulmonary arterial pressure/ systolic systemic arterial pressure (sPAP)/sSAP) ratio groups. Error bars: interquartile range (IQR). * indicates *P*‐value < 0.05.

In 2009, we established TAPSE as a valuable echocardiographic measure of systolic RV function in childhood.^4^ In recent years, TAPSE has evolved into one of the major clinical indicators of systolic longitudinal RV function in pediatric and adult PH patients.^1^ The most commonly employed method for noninvasive estimation of (elevated) systolic PAP is the Doppler measurement of TRV, as an estimation of the systolic pressure difference between the RV and the right atrium. TAPSE is vastly independent of heart rate and can be determined even at high heart rate, making this echocardiographic variable especially suitable for children.^1^ In particular, TAPSE is an accepted means to measure longitudinal systolic RV function in children.^1^

This is the first pediatric study on this specific combinatory echocardiographic parameter approach, i.e., the TRV/TAPSE ratio (m/s:cm), in a mid‐sized cohort of children with PH. We have since found that the TRV/TAPSE ratio might be associated with worsening NYHA FC in our pediatric PH patients. In our experience, TAPSE values become abnormally low in the later stages of PH/hypertensive pulmonary vascular disease, which may explain its good, inverse correlation with clinical outcome;^1^ however, TAPSE is probably not very sensitive in detecting mild to moderate RV systolic function in the earlier stages of PH. Current research on noninvasive imaging has started to combine the information provided by TAPSE (longitudinal systolic RV function) with indicators of RV pressure afterload, such as the TRV. In adults, the clinical endpoint was shown to be more frequent in patients with a TRV/TAPSE ratio >4.5 than with TRV/TAPSE ratio ≤4.5.^5^ Determining the TRV/TAPSE ratio avoids overreliance on a single parameter, and as such is in line with other multi‐parametric strategies in pediatric echocardiography.^2^Based on the results of our current report, the TRV/TAPSE ratio has the potential to become an echocardiographic surrogate measure for RV‐PA coupling. But caution should be employed when using echocardiographic parameters as surrogates for hemodynamic performance as they have not yet been validated against invasive pressure‐volume loop‐derived measurements of RV‐PA coupling.

A major limitation of this preliminary study is that because of the small sample size, our novel TRV/TAPSE ratio could not be sufficiently compared to other prognostic variables. Therefore, future (multicenter) studies are warranted to provide sufficient statistical prognostic relevance of this novel parameter.

In summary, we found that the TRV/TAPSE ratio increased with NYHA FC in children with PH. In future, multicenter studies related to PH and the TRV/TAPSE ratio maybe established as a potential parameter of clinical outcome.

## Disclosure

GH currently receives grant support from the German Research Foundation (DFG; HA 4348/6‐1, KFO311). The authors declare no conflict of interest.

## Author contributions

M.K. is the main author and wrote the manuscript; A.A. and K.M. collected and analyzed data; M.C. drafted the manuscript and G.H. critically reviewed the manuscript and supervised the study process. All authors read and approved the final manuscript. 

## Supporting information


**Table S1** Demographic data.
