# Supplementary material for: A novel echocardiographic approach indicates disease severity in pediatric pulmonary hypertension
Source: Pediatr Int. 2020 Apr 17;62(5):637–9. doi: 10.1111/ped.14163 (PMC7318688; doi:10.1111/ped.14163)
Supplement: Supplementary file 1 — Table S1 Demographic data. [file PED-62-637-s001.docx]

| **Table 1 - Demographic Data** | | |  |  |
| --- | --- | --- | --- | --- |
| **PH Patients** |  | |  | n or median  (range or IQR) |
|  |  | | n | 47 |
|  | Female | | n (%) | 22 (46.8) |
|  | Age at baseline (years) | | median (range) | 7.2 (0.4 – 18) |
|  | Body Weight (kg) | | median (range) | 21.7 (5.5 – 73.7) |
|  | Body Length (cm) | | median (range) | 130 (49 - 189) |
|  | BSA (m²) | | median (range) | 0.87 (0.36 – 1.98) |
|  | PAH-CHD | | n | 30 |
|  | IPAH | | n | 8 |
|  | PH-BPD | | n | 9 |
|  |  | |  |  |
|  | NYHA-FC/ROSS score: | |  |  |
|  | I | | n | 18 |
|  | II | | n | 20 |
|  | III | | n | 9 |
|  | *Stratification by PH severity* | |  |  |
|  | sPAP/sSAP ratio: | |  |  |
|  | < 0.5 | (group 1) | n | 8 |
|  | 0.5–0.8 | (group 2) | n | 21 |
|  | > 0.8 | (group 3) | n | 18 |
| **Medication** |  | |  |  |
|  | Bosentan (mono) | | n | 2 |
|  | Bosentan + Sildenafil | | n | 5 |
|  | Macitentan (mono) | | n | 8 |
|  | Macitentan + Sildenafil | | n | 13 |
|  | Sildenafil (mono) | | n | 12 |
|  | Calcium channel blockers | | n | 3 |
|  | Selexipag | | n | 4 |
| **Hemodynamics** |  | |  |  |
|  | sPAP/sSAP | | % (IQR) | 71 (55 – 91) |
|  | mPAP (mmHg) | | median (IQR) | 39 (32 – 48) |
|  | PVRi | | median (IQR) | 6.2 (3.7 – 9.2) |
|  | TRV/TAPSE ratio (m/s:cm) | | median (IQR) | 2.64 (2.24 – 3.46) |
| **Echocardiography** |  | | median (IQR) |  |
|  | TRV (m/s) | | median (IQR) | 4.0 (3.5 – 4.3) |
|  | TAPSE (cm) | | median (IQR) | 1.52 (1.21 – 1.74) |
|  | TAPSE z-score | | median (IQR) | -2.79 (-3.67 – -1.78) |

**Legend to Supplemental Table**

Demographic Data of our PH patients. Age of our patients at baseline is the age at inclusion in the study. Subgroups of patients (PAH-CHD, IPAH, PH-BPD) are provided. *Abbreviations*: Body surface area (BSA); idiopathic PAH (IPAH); mean pulmonary artery pressure (mPAP); New York Heart Association (NYHA); pulmonary hypertension (PH); PAH associated with congenital heart disease (PAH-CHD); PH secondary to bronchopulmonary dysplasia (PAH-BPD); indexed pulmonary vascular resistance (PVRi); RV, right ventricle; systolic pulmonary artery pressure (sPAP); systolic systemic artery pressure (sSAP); tricuspid regurgitation velocity (TRV), tricuspid annular plane systolic excursion (TAPSE).
